# 

**Published:** 1903-03

**Authors:** 


### THE BRISTOL

### Medico-Chirurgical Journal.

A JOURNAL OF THE MEDICAL SCIENCES FOR THE WEST OF ENGLAND AND SOUTH WALES

*PUBLISHED UNDER THE AUSPICES OF*

*THE BRISTOL MEDICO-CHIRURGICAL SOCIETY.*

EDITOR:

R. SHINGLETON SMITH, M.D., B.Sc.,

WITH WHOM ARE ASSOCIATED

J. MICHELL CLARKE, M.A., M.D., J. LACY FIRTH, M.S., M.D., AND JAMES SWAIN, M.S., M.D.

ASSISTANT-EDITOR:

P. WATSON WILLIAMS, M.D.

EDITORIAL SECRETARY:

JAMES TAYLOR.

"Scire est nescire, nisi id me Scire alius sciret."

LIBRARY

SURGEON GENERAL'S OFFICE

NOV.-9-1904

185373.

*VOL. XXI.*

BRISTOL: J. W. ARROWSMITH.

LONDON: J. & A. CHURCHILL, 7 GREAT MARLBOROUGH STREET.

1903.

Wi
BR323

J. W. Arrowsmith Publisher Bristol

33756

### CONTENTS OF VOLUME XXI.

Page

Therapeutics of a Sea Voyage. By R. ROXBURGH, M.B., F.R.C.S. Ed. 1

On the Probable Rhythmical Contraction of the Bronchial Muscular Coat as a Factor in Pulmonary Disease. By P. WATSON WILLIAMS, M.D. Lond. ... ... ... ... ... ... 6

A Case of Banti's Disease, with Autopsy. By J. MICHELL CLARKE, M.A., M.D. Cantab., F.R.C.P. ... ... ... ... ... 14

Remarks on the "Light Treatment" of Lupus Vulgaris and Erythematosus, and Rodent Ulcer, &c., as carried out at the Bristol General Hospital since December 9th, 1901, to the end of December, 1902. By A. J. HARRISON, M.B. Lond., and W. KENNETH WILLS, M.A., M.B., B.C. Cantab. ... ... 23

Perforated Typhoid Ulcer. By H. G. KYLE, M.B., B.Ch. Oxon. ... 30

On Some of the Initial Signs of Pleurisy. By F. H. EDGEWORTH, M.B., B.A. Cantab., D.Sc. Lond. ... ... ... ... ... 36

Two Cases of Ruptured Tubal Pregnancy. By HAROLD F. MOLE, F.R.C.S. ... ... ... ... ... ... ... ... ... 39

Mobility. By JOHN CHIENE, C.B., LL.D., F.R.S.E., M.D. ... ... 97

The Influence of the Auricles on the Percussion of the Heart. By D. R. PATERSON, M.D., M.R.C.P. ... ... ... ... ... 110

Some Remarks upon the Finsen Light Treatment of Lupus. By W. KENNETH WILLS, M.A., M.B., B.C. Cantab., M.R.C.S. ... 119

On the Diagnosis of Malignant Disease of the Body of the Uterus, with Notes of a Case treated twice by Curettage, and finally by Hysterectomy. By ERNEST W. HEY GROVES, M.D., B.Sc. Lond. 128

Missing Links in Joint Disease. By PRESTON KING, B.A., M.D., B.C. Cantab. ... ... ... ... ... ... ... ... 137

Joseph Griffiths Swayne, M.D. Lond. ... ... ... ... ... 193

Round the World. A Medical Traveller's Notes. By R. ROXBURGH, M.B., F.R.C.S. Ed. ... ... ... ... ... ... ... 202

A Contribution to the Conservative Surgery of the Uterine Appendages. By JAMES SWAIN, M.S., M.D. Lond., F.R.C.S. ... 211

A Case of Perforated Duodenal Ulcer, with Remarks. By G. MUNRO SMITH. ... ... ... ... ... ... ... ... ... 218

The Antibodies in Disease. By F. BUSHNELL, M.D. Lond. ... 221

CONTENTS.

Page

A Granuloma of the Nose, due to Iodide of Potassium. By C. Percival Crouch, M.B. Lond., F.R.C.S. .. ... ... ... 231

The Practical Value of Blood-Counts. By William Gordon, M.A., M.D. Cantab. ... ... ... ... ... ... ... ... 234

Four Cases of Renal Calculi treated successfully by Lumbar Nephro-Lithotomy. By E. J. Trevor Jones, M.D. Brux., M.R.C.S., L.R.C.P. ... ... ... ... ... ... ... ... ... 245

Some Surprises in my Professional Career. By J. Paul Bush, C.M.G. 289

Light Treatment. By A. J. Harrison, M.B. Lond., and W. Kenneth Wills, M.A., M.B., B.C. Cantab. ... ... ... 303

The Dangers of Delay in Ovariotomy. By T. Carwardine, M.S., M.B. Lond., F.R.C.S. ... ... ... ... ... ... ... 309

A Case of Infantile Scurvy. By Bertram M. H. Rogers, M.D., B.Ch., B.A. Oxon. ... ... ... ... ... ... ... 318

Note on Scurvy as a Cause of Hæmaturia in the Infant and in Old Age. By R. Shingleton Smith, M.D., B.Sc. Lond. ... ... 320

A Case of Infantile Scurvy or Scurvy-Rickets. By Christopher Elliott, B.A., M.D. ... ... ... ... ... ... ... 323

Trypanosomiasis. By J. Odery Symes, M.D., D.P.H. Lond. ... 325

Muscular Atrophy and Sclerodermia. By J. A. Nixon, M.B., B.C. Cantab., M.R.C.P. Lond. ... ... ... ... ... .. 328

Collargol: A Review of Some of its Clinical Applications, with Experiments on its Antiseptic Action. By J. M. Fortescue-Brickdale, M.A., M.D. Oxon. ... ... ... .. ... 337

Progress of the Medical Sciences ... ... 44, 143, 248, 345

Reviews of Books .. ... ... ... ... 54, 158, 260, 358

Editorial Notes ... ... ... ... ... 71, 165, 279, 369

Notes on Preparations for the Sick ... ... 79, 173, 281, 373

The Library of the Bristol Medico-Chirurgical Society ... ... ... ... 81, 176, 284, 377

Meetings of Societies ... ... ... ... ... 83, 179, 379

Obituary ... ... ... ... ... ... ... 94, 187, 285

Local Medical Notes ... ... ... ... 95, 192, 287, 383

### The Library of the Bristol Medico-Chirurgical Society.

*The following donations have been received since the publication of the List in December:*

*February 28th, 1903.*

| | |
|---|---|
| Boston Medical Library (1) ... ... ... ... ... | 10 volumes |
| The General Medical Council (2) ... ... ... ... | 1 volume |
| L. M. Griffiths (3) ... ... ... ... ... ... ... | 1 ,, |
| Medical and Chirurgical Faculty of the State of Maryland (4) ... ... ... ... ... ... ... | 1 ,, |
| Pathological Society of London (5) ... ... ... | 1 ,, |
| Society for the Study of Disease in Children (6) ... | 1 ,, |
| The Surgeon-General, United States Army (7) ... | 1 ,, |

### FORTY-SEVENTH LIST OF BOOKS.

The titles of books mentioned in previous lists are not repeated.

The figures in brackets refer to the figures after the names of the donors, and show by whom the volumes were presented The books to which no such figures are attached have either been bought from the Library Fund or received through the *Journal*.

| | | |
|---|---|---|
| **Bell, R.** ... ... | *Cancer*... ... ... ... ... ... ... ... ... ... ... ... | 1903 |
| *Catalogue (Index) of the Library of the Surgeon-General's Office, United States Army* | (7) 2nd Ser. Vol. VII. | 1902 |
| **Crocker, H. R.** . . | *Diseases of the Skin.* 2 vols.... ... ... ... ... 3rd Ed. | 1903 |
| **Delamotte, W. A.** | *An Historical Sketch of St. Bartholomew's Hospital* ... | 1844 |
| **Dickinson, R. C. Norris and R. L.** | *A Text-Book of Obstetrics.* 2 vols. 2nd Ed. | 1902 |
| **Fenwick, S. and W. S.** | *Cancer and Tumours of the Stomach* ... ... ... | 1902 |
| **Fox, G. H.** ... | *Photographic Atlas of the Diseases of the Skin* ... ... | 1902 |
| **Goadby, K. W.** ... | *The Mycology of the Mouth* ... ... ... ... ... ... | 1903 |
| **Gould, G. M.** ... | *Biographic Clinics* ... ... ... ... ... ... ... ... ... | 1903 |
| **Hall, I. W.** ... | *The Purin Bodies of Food Stuffs* ... .. ... ... ... | 1902 |
| **Harrison, R.** ... | *The Ambulance in Civil Life on Land and Sea.* 5th Ed. | 1902 |
| **Haultain, F. W. N.** | *A Practical Handbook of Midwifery* ... ... 2nd Ed. | [N.D.] |
| **Helferich, H.** ... | *Atlas and Epitome of Traumatic Fractures and Dislocations* (Edited by J. C. Bloodgood) ... 5th Ed. | 1902 |
| **Henderson, E.** ... | *The Nurse in Hot Climates* ... ... ... ... ... ... | 1903 |
| **Herman, G. E.** ... | *Diseases of Women* ... ... ... Revised [2nd] Ed. | [N.D ] |
| **Hewer, Mrs. J. L.** | *Our Baby* ... ... ... ... ... ... ... ... ... 8th Ed. | 1902 |
| **Kuhn, P.** ... ... | *Inoculation against Malaria* (Tr. by H. A. Nesbitt) ... | 1902 |

**Latham, A.** ... *Pulmonary Consumption* ... ... ... ... ... ... ... ... 1903
**Mackenzie, J.** ... *The Study of the Pulse* ... ... ... ... ... ... ... ... 1902
**Maddox, E. E.** ... *Golden Rules of Refraction* ... ... ... ... ... ... ... [N.D.]
**Moullin, C. M.** ... *The Surgical Treatment of Ulcer of the Stomach* ... ... 1902
**Norris and R. L. Dickinson, R. C.** *A Text-Book of Obstetrics.* 2 vols. 2nd Ed. 1902
**Scudder, C. L.** ... *The Treatment of Fractures* ... ... ... ... 3rd Ed. 1902
**Smith, G.** [Ed.].. *Physician and Friend—Alexander Grant, F.R.C.S.* ... 1902
**Strange, W.** ... *The Seven Sources of Health* ... ... ... ... ... (3) 1864
**Taylor, M. L.** ... *Second Progress Report of the Campaign against Mosquitoes in Sierre Leone* ... ... ... ... ... 1902
,, ... *Report on the Sanitary Conditions of Cape Coast Town*... 1902

### TRANSACTIONS, REPORTS, JOURNALS, &c.

American Journal of Obstetrics, The (1) Vols. XIV., 1881; XXIII., 1890; XXV. 1892
Archives de Neurologie ... ... ... ... ... .. Tomes XIII., XIV. 1902
Association of American Physicians, Transactions of the. Vol. XVII. 1902
Boston Medical and Surgical Journal, The (1) Vols. LXVII., 1863; XC., 1874; XCII.—XCV., 1875-76; XCIX. 1878
British Almanac, The ... ... ... ... ... ... ... ... ... ... ... 1903
British Medical Journal, The ... ... ... ... ... ... ... Vol. II. for 1902
Bulletin de l'Académie de Médicine de Paris. Tomes XLV., XLVI. 1901
Catalogue of Current Literature, The Reference ... ... ... 2 parts 1902
Clinical Journal, The ... ... ... ... ... ... ... ... Vol. XX. 1902
Echo medical du Nord, L' ... ... ... ... ... ... ... [Vol. VI.] 1902
Edinburgh Medical Journal, The ... ... ... ... ... N.S., Vol. XII. 1902
General Medical Council, Minutes of the ... ... .. (2) Vol. XXXIX. 1903
Glasgow Medical Journal, The ... ... ... ... ... ... Vol. LVIII. 1902
Hazell's Annual ... ... ... ... ... ... ... ... ... ... ... ... ... 1903
Hospitals—
- Guy's Hospital Reports ... ... ... ... ... ... Vol. LVII. 1902
- Johns Hopkins Hospital Reports, The Vol. X., Nos. 6-7-8-9 1902
- St. Bartholomew's Hospital Reports ... ... ... Vol. XXXVIII. 1903

Journal of Cutaneous and Genito-Urinary Diseases ... ... Vol. XX. 1902
Lancet, The ... ... ... ... ... ... ... ... ... ... Vol. II. for 1902
Medical and Chirurgical Faculty of the State of Maryland, Transactions of the ... ... ... ... ... ... ... ... ... ... ... ... (4) [1902]
Medical Chronicle, The ... .. ... ... ... ... 4th Ser. Vol. III. 1902
Medical Directory, The ... ... ... ... ... ... ... ... ... ... ... 1903
Medical Press and Circular, The ... ... ... ... ... [Vol. CXXV.] 1902
Medical Record ... ... ... ... ... ... ... ... ... ... Vol. LXII. 1902
Medical Society of London, Transactions of the ... ... Vol. XXV. 1902
Medico-Chirurgical Transactions ... ... ... ... ... Vol. LXXXV. 1902
New Hampshire Medical Society, Transactions of the ... ... ... ... 1902
Odontological Society of Great Britain, Transactions of the Vol. XXXIV. 1902
Otological Society of the United Kingdom, Transactions of the Vol. III. 1902
Pathological Society of London, Transactions of the (5) Vol. LIII. 1902
Public Health ... ... ... ... ... ... ... ... ... ... Vol. XIV. [1902]

Quarterly Medical Journal, The ... ... ... ... ... ... ... Vol. X. 1902

Révue de Thérapeutique médico-chirurgicale ... ... ... ... ... ... 1902

Scottish Medical and Surgical Journal, The ... ... ... ... Vol. XI. 1902

Smithsonian Report, 1901 ... ... ... ... ... ... ... ... ... ... 1902

Society for the Study of Disease in Children, Reports of the (6) Vol. II. 1901-2

Univ. of Penna. Medical Bulletin ... ... ... ... ... ... ... Vol. XIV. 1902

West London Medical Journal ... ... ... ... ... ... Vol. VII. 1902

Whitaker's Almanack ... ... ... ... ... ... ... ... ... ... ... ... 1903

Who's Who ... ... ... ... ... ... ... ... ... ... ... ... ... 1903

### MEETINGS OF SOCIETIES.

### Bristol Medico-Chirurgical Society.

### Local Medical Notes.

**Bristol Royal Infirmary.**—Mr. W. T. Mc Cowen, M.R.C.S., L.R.C.P., has been appointed Junior House Surgeon and Anæsthetist. Mr. J. H. S. Hawes, M.B., B.S. (Dunelm), has been appointed Casualty Officer.

**University College, Bristol.**—Dr. P. Watson Williams, M.D. Lond., has been appointed Lecturer on Practical Medicine.

Messrs. K. H. Beverley and F. C. Whitmore have obtained the M.R.C.S. and L.R.C.P. diplomas. Mr. C. G. Russ Wood, of Shrewsbury, a former Bristol student, has obtained the F.R.C.S. Eng. diploma. Mr. F. P. Mackie, F.R.C.S. Eng., L.R.C.P., in the last Indian Medical Service Examination obtained the first place and the Montefiore Prize of twenty guineas.

**The Bristol General Hospital.**—Mr. W. S. V. Stock, M.B., B.S. Lond., M.R.C.S., has been appointed House Surgeon to the Hospital. Mr. A. F. Martin, M.B., Ch.B., Vict., late House Surgeon to the Hospital, has been appointed House Surgeon to the North Staffordshire Infirmary.

**Association of Midland Railway Surgeons.**—At a meeting of Midland Railway Surgeons, held in Birmingham on November 27th last, it was decided to form the above Association on lines similar to those of the long-established Great Western Railway Provident Society Medical Staff. Any Midland Railway Surgeons wishing to join the Association should send their names and addresses to Dr. Ffennell MacCarthy, 58 The Tything, Worcester.

Zymotic Diseases.—The editor has to thank Dr. D. S. Davies for the following notes on the various zymotic diseases prevalent in the city of late:—

1. *Smallpox.*—This disease has recently been somewhat prevalent in many of the Midland and North Country towns, with a tendency to spread southwards, often through tramps.

No smallpox has been introduced into Bristol from October, 1902, when a fatal case occurred in a coloured sailor arriving on a steamship from Salonika, until early in January, 1903, when a girl of 16, living in the middle of the city, developed smallpox. The origin of this case was obscure, but was probably through some articles of clothing received from relatives in infected districts. Two resultant cases—one in an unvaccinated sister, the other in a "devaccinated"[1] woman of 35, who refused re-vaccination, completed the history of this introduction.

At the end of January a tramp at Horfield prison developed

[1] I use the term "devaccinated" with Dr. Bond's meaning; viz., vaccinated in infancy, but having outlived the protection period."

smallpox, and was removed to the city hospital at Novers Hill; no resulting cases followed.

On February 12th, a youth at Avonmouth, who had visited Cardiff for a week, developed smallpox at the correct period to point to infection received in Cardiff.

2. *Scarlet Fever.*—This disease has now been prevalent in epidemic form for three years, and the usual remission may be expected soon. Although the attack rate on the population is the highest recorded since Notification commenced in 1890, it is satisfactory to note that the death-rate has not risen proportionately, and has been exceeded in two recent epidemics; viz., in 1892 and 1896. The disease is of fairly mild type, the case mortality per cent. being 2.4, which has been exceeded on eight occasions in the past thirteen years.

The actual number of deaths has been 66, out of a total population of 338,000, which compares most favourably with the terrible mortality from this disease forty years ago. In the 1863–64 epidemic, over 1,100 deaths occurred from scarlet fever alone, although the population then numbered only some 160,000 persons.

*Diphtheria.*—This disease has proved a source of far greater anxiety than either smallpox or scarlet fever since February of 1900, when the virulent, rapidly spreading form first made its appearance in the southern portion of the city, in Bedminster. Between the years 1886 and 1900 diphtheria had never proved a source of trouble or proved difficult to control; it caused no school outbreaks, and the death rate over a period of 10 years was less than half that recorded for the large towns of England and Wales.

But since 1900 it has invaded district after district, showing high infectivity, and causing considerable mortality. It has thus not only thrown considerable strain upon the exiguous city accommodation for infectious diseases, but has also pressed heavily upon the local Hospital and Infirmary, to which urgent cases have been sent for operative relief.

The efficient control of this disease in virulent form can only be secured by constant medical examination of contacts in infected houses, who so frequently act as "carriers," harbouring the bacilli in nose or throat without obvious clinical symptoms, and in this way securing its persistence. The colossal nature of this work, with the vast amount of bacteriological examination and re-examination necessary, has as yet been grasped by few local authorities, for the majority cannot understand the existence of a disease that is not to be controlled by isolation, disinfection, and attention to drains.

4. *Measles.*—During the autumn this disease was very prevalent and fatal. The school attendance was considerably affected, and a system of school notification adopted, so as to give some opportunity of visiting and cautioning the inmates of affected houses. The effect, however, was not very marked, and the disease burnt itself out as usual.

